# Comparative analysis of the performance of the large language models ChatGPT-3.5, ChatGPT-4 and Open AI-o1 in the field of Programmed Cell Death in myeloma

**DOI:** 10.1007/s12672-025-02648-3

**Published:** 2025-05-23

**Authors:** Wu Kun, Tao Bo, Li Yuntao, Cheng Shenju, Li Yanhong, Luo Shan, Zeng Yun, Nie Bo, Shi Mingxia

**Affiliations:** 1 Yunnan Key Laboratory of Laboratory Medicine, Yunnan Province Clinical Research Center for Laboratory Medicine, Department of Clinical Laboratory, The First Affiliated Hospital of Kunming Medical University, Kunming, 650032 China; 2 Information Center, The First Affiliated Hospital of Kunming Medical University, Kunming, 650032 China; 3 Department of Hematology, Hematology Research Center of Yunnan Province, the First Affiliated Hospital of Kunming Medical University, Kunming, 650000 China

**Keywords:** Programmed cell death, Multiple myeloma, Large language models, Clinical decision support, Accuracy

## Abstract

**Objective:**

This study aimed to compare the performance of three large language models (LLMs)—ChatGPT-3.5, ChatGPT-4, and Open AI-o1—in addressing clinical questions related to Programmed Cell Death in multiple myeloma. By evaluating each model's accuracy, comprehensiveness, and self-correcting capabilities, the investigation sought to determine the most effective tool for supporting clinical decision-making in this specialized oncological context.

**Methods:**

A comprehensive set of forty clinical questions was curated from recent high-impact oncology journals, International Myeloma Working Group (IMWG) guidelines, and reputable medical databases, covering various aspects of Programmed Cell Death in multiple myeloma. These questions were refined and validated by a panel of four hematologists-oncologists with expertise in the field. Each question was individually posed to ChatGPT-3.5, ChatGPT-4, and Open AI-o1 in controlled sessions. Responses were anonymized and evaluated by the same panel using a five-point Likert scale assessing accuracy, depth, and completeness. Responses were categorized as “excellent,” “satisfactory,” or “insufficient” based on cumulative scores. Additionally, the models’ self-correcting abilities were assessed by providing feedback on initially insufficient responses and re-evaluating the revised answers. Interrater reliability was measured using Cohen’s Kappa coefficients.

**Results:**

Open AI-o1 consistently generated the most extensive and detailed responses, achieving significantly higher total scores across all domains compared to ChatGPT-3.5 and ChatGPT-4. It demonstrated the lowest proportion of "insufficient" responses and the highest percentage of “excellent” answers, particularly excelling in guideline-based questions. Open AI-o1 also exhibited superior self-correcting capacity, effectively enhancing its responses upon receiving feedback. The highest Cohen’s Kappa coefficient among the models indicated greater consistency in evaluations by clinical experts. User satisfaction surveys revealed that 85% of hematologists-oncologists rated Open AI-o1 as "highly satisfactory," compared to 60% for ChatGPT-4 and 45% for ChatGPT-3.5.

**Conclusion:**

Open AI-o1 outperforms ChatGPT-3.5 and ChatGPT-4 in accuracy, depth, and reliability when addressing complex clinical questions related to Programmed Cell Death in multiple myeloma. Its advanced “thinking” ability facilitates comprehensive and evidence-based responses, making it a more dependable tool for clinical decision support. These findings suggest that Open AI-o1 holds significant potential for enhancing clinical practices in specialized oncological fields, though ongoing validation and integration with human expertise remain essential.

## Introduction

Multiple myeloma, a hematological malignancy characterized by the clonal proliferation of plasma cells, continues to pose significant clinical challenges despite advances in therapeutic strategies [1–3]. A pivotal aspect of its pathophysiology is Programmed Cell Death, wherein myeloma cells adapt their metabolic pathways to sustain rapid growth, evade apoptosis, and resist therapeutic interventions [4–6]. Understanding the intricate metabolic alterations in multiple myeloma is crucial for the development of targeted therapies and improving patient outcomes [7, 8]. However, the complexity and dynamic nature of metabolic networks necessitate sophisticated analytical tools to synthesize and interpret vast amounts of biomedical data effectively [9–11].

In recent years, large language models (LLMs) such as ChatGPT-3.5, ChatGPT-4, and the more recent Open AI-o1 have emerged as transformative technologies with the potential to revolutionize various domains, including healthcare. These models, developed using advanced machine learning algorithms and extensive datasets, are capable of generating coherent, contextually relevant, and information-rich responses to a wide array of queries [12–14].Although the exact composition of these models’ training corpora remains proprietary, preliminary documentation suggests that ChatGPT-3.5 and ChatGPT-4 were primarily trained on broad, internet-scale datasets encompassing diverse topics, while Open AI-o1 integrated additional specialized medical and oncological resources as part of its dataset. This integration of more domain-specific material may underlie Open AI-o1’s superior performance in clinical oncology queries.However, as the complexity of clinical inquiries increases, particularly in specialized fields like metabolic oncology, the limitations of these models become more apparent. Issues such as contextual misunderstanding, incomplete data integration, and the inability to perform deep analytical reasoning can hinder their effectiveness in providing precise and actionable medical insights. Enter Open AI-o1, the latest advancement in the lineage of OpenAI’s language models. Released in December 2024, o1 distinguishes itself from its predecessors through its enhanced “thinking” capability, which allows it to engage in more complex reasoning tasks before generating responses [15–17]. This iterative "chain of thought" process enables o1 to dissect intricate scientific concepts, perform detailed analyses, and provide more accurate and comprehensive answers, particularly in fields demanding high levels of expertise such as Programmed Cell Death in multiple myeloma. Unlike ChatGPT-4, which primarily relies on pattern recognition and data synthesis from its training corpus, o1 incorporates a novel optimization algorithm and reinforcement learning techniques that enhance its reasoning prowess and adaptability to nuanced clinical scenarios [18–21]. The development of o1 reflects a significant paradigm shift in AI model architecture, moving beyond mere data processing to embody a more sophisticated form of cognitive simulation [22–24]. This advancement is not merely incremental but represents a foundational enhancement that improves the model's ability to handle complex scientific inquiries, thereby bridging the gap between artificial intelligence and human-like critical thinking [25–27]. Early benchmarks and performance assessments have positioned o1 as superior to ChatGPT-4 in tasks requiring deep scientific reasoning, mathematical problem-solving, and the integration of multifaceted biomedical information. Such capabilities are particularly pertinent to the field of metabolic oncology, where understanding the interplay between various metabolic pathways and their implications for disease progression and treatment resistance is essential.

In the context of multiple myeloma, where Programmed Cell Death plays a crucial role in disease pathogenesis and therapeutic resistance, the ability to accurately and comprehensively analyze and interpret metabolic pathways is invaluable [28–30]. Traditional methods of data synthesis and literature review are time-consuming and may not keep pace with the rapid advancements in the field. LLMs like ChatGPT-3.5, ChatGPT-4, and Open AI-o1 offer a promising solution by automating the synthesis of complex information, thereby accelerating research and clinical decision-making processes.

This study aims to fill this knowledge gap by conducting a comparative analysis of ChatGPT-3.5, ChatGPT-4, and Open AI-o1 in addressing clinical questions related to Programmed Cell Death in multiple myeloma. By evaluating each model’s performance in terms of accuracy, depth, comprehensiveness, and self-correcting capacity, the investigation seeks to elucidate the strengths and limitations of these LLMs in a specialized oncological context.

## Methods

### Study design

A comprehensive set of clinical inquiries pertaining to Programmed Cell Death in multiple myeloma was curated by reviewing recent publications in high-impact oncology journals, guidelines from the International Myeloma Working Group (IMWG), and reputable oncology-focused medical databases. This process resulted in the compilation of forty clinical questions, encompassing various aspects such as the underlying metabolic pathways, diagnostic biomarkers, therapeutic targets, resistance mechanisms, and prognostic indicators in multiple myeloma. To ensure relevance and clinical applicability, these questions were further refined by a panel of four hematologists-oncologists with specialized expertise in multiple myeloma and metabolic oncology. This expert panel validated the questions for their pertinence to current clinical practices and research advancements in the field.To finalize the list of forty questions, the panel conducted a systematic review to identify and remove any duplicates or near-duplicates, ensuring that each item addressed a distinct clinical or research aspect of Programmed Cell Death in multiple myeloma. This process involved cross-referencing the draft question set against existing literature and each expert’s knowledge base, ultimately excluding three items due to substantial overlap. Additionally, the panel employed a reproducible screening framework—focusing on clinical relevance, novelty, and guideline-based importance—to refine the pool of questions derived from journals and IMWG guidelines. No question was excluded from the final analysis solely on the basis of the models’ inability to generate an initial answer; in cases where an answer was completely missing, the question was retained to assess how the model would respond upon a second prompt or clarification.This study was reviewed by the Yunnan Province Clinical Research Center for Laboratory Medicine Ethics Committee, which concluded that no ethical approval was required to conduct the study.

### Accuracy assessment

The performance of ChatGPT-3.5, ChatGPT-4, and Open AI-o1 was evaluated by presenting each of the forty questions individually to the respective language models in separate, controlled sessions. All three models received the exact same set of input prompts, with no alterations in wording, to ensure a fair comparison of their outputs. The default settings for each system—such as temperature, maximum tokens, and other model parameters—were left unchanged, aligning with the manufacturers’ recommended configurations for standard usage. Importantly, the models were not provided with any external reference materials, guidelines, or articles beyond the prompt itself; the questions were presented in a purely open-ended fashion to evaluate each model’s baseline knowledge.The same panel of four hematologists-oncologists who assessed the accuracy of each response also participated in the user satisfaction surveys, ensuring consistency in evaluating both the technical and practical aspects of the models’ performances. Prior to each query, the conversation context was cleared to prevent any influence from previous interactions. The responses generated by each model were transcribed into plain text, ensuring anonymity by removing any indicators of the originating model. Four independent hematologists-oncologists, blinded to the identity of the language models, assessed each response based on a predefined evaluation framework. This framework employed a five-point Likert scale, where a score of 1 denoted a completely inaccurate response, 2 indicated significant inaccuracies, 3 represented partial correctness with some missing details, 4 signified accurate responses with adequate detail, and 5 reflected comprehensive and highly accurate answers. The cumulative score for each response was the aggregate of the four reviewers' ratings. Responses scoring above 16 were categorized as "excellent," scores between 12 and 16 as "satisfactory," and scores below 12 as "insufficient." Interrater reliability was quantified using Cohen’s Kappa coefficients to determine the consistency among the reviewers' evaluations.

### Re-evaluating self-correcting capacity

Responses initially deemed "insufficient" underwent a secondary evaluation to assess the models' ability to self-correct. For each inadequate response, detailed feedback highlighting specific inaccuracies or omissions was provided to the respective language model. This feedback was standardized to include a concise explanation of the primary error or omission (e.g., lack of a guideline reference or misinterpretation of a biomarker) without offering excessively detailed “hints” or direct citations. The intent was to allow each model enough information to recognize and correct its mistakes while minimizing any undue bias introduced by the reviewers. If a model generated references or citations not found in recognized databases (i.e., “hallucinated” references), these were flagged as inaccurate, and the model was instructed to verify its sources. Any revised response that retained unverifiable references was still judged as insufficient. This procedure ensured that all corrections derived from the model’s internal reasoning processes rather than from extensive external guidance.The models were then prompted to generate revised answers incorporating the suggested corrections. These revised responses were again anonymized and evaluated by the same panel of hematologists-oncologists, who were unaware of the initial assessments. The improvement in scores from the initial to the revised responses was documented to gauge the efficacy of each model's self-correcting mechanisms.

### Statistical analysis

Data derived from the expert evaluations were systematically organized and analyzed using the statistical software R (version 4.2.0). Descriptive statistics were calculated to summarize the mean and standard deviation for normally distributed variables, and median with interquartile ranges for non-normally distributed data. Comparative analyses between the three language models were performed using ANOVA for normally distributed continuous variables and the Kruskal–Wallis test for non-parametric data. Post hoc pairwise comparisons with appropriate adjustments were conducted to identify significant differences between specific models. These thresholds align with the commonly referenced Landis and Koch criteria. Specifically, “moderate” corresponds to a coefficient in the 0.41–0.60 range, “substantial” denotes 0.61–0.80, and “almost perfect” reflects 0.81–1.00. A p-value of less than 0.05 was considered statistically significant, ensuring the robustness of the comparative findings.

## Results

A total of forty clinical questions related to Programmed Cell Death in multiple myeloma were administered to ChatGPT-3.5, ChatGPT-4, and Open AI-o1. These questions encompassed various domains, including metabolic pathways, diagnostic biomarkers, therapeutic targets, resistance mechanisms, and prognostic indicators. An additional thirty questions, derived from the latest guidelines of the International Myeloma Working Group (IMWG) and recent high-impact oncology publications, were also presented to the models. Four independent hematologists-oncologists evaluated each response, and interrater reliability was assessed using Cohen’s Kappa coefficients across all three large language models (LLMs).

### Length of responses from LLM chatbots

Table 1 summarizes the average word and character counts generated by each model in response to general inquiries about Programmed Cell Death in multiple myeloma. Open AI-o1 consistently produced the most extensive responses, averaging 450.67 ± 60.45 words, which was significantly higher than ChatGPT-3.5 at 320.50 ± 55.30 words (p < 0.01) and ChatGPT-4 at 390.25 ± 50.80 words (p < 0.05). A similar trend was observed in character counts, with Open AI-o1 generating an average of 28,589.65 ± 3,200.62 characters, surpassing ChatGPT-3.5’s 20,254.40 ± 2,800.66 characters (p < 0.01) and ChatGPT-4’s 24,758.30 ± 2986.85 characters (p < 0.05). Responses to guideline-derived questions (Table 2) revealed that ChatGPT-3.5 produced shorter answers, with a median word count of 350.00 (300.00–400.00), compared to Open AI-o1’s median of 500.00 (450.00–550.00) words (p < 0.01). ChatGPT-4 generated responses with a median of 420.00 (380.00–460.00) words, which was significantly less than Open AI-o1 (p < 0.05) but more than ChatGPT-3.5. Character counts followed a similar pattern, indicating that Open AI-o1 consistently delivers more comprehensive textual outputs.

**Table 1:** Length of LLMs‑chatbots’ responses to general Programmed Cell Death questions

Response Length (Words)	Response Length (Characters)	character counts (Characters)
ChatGPT-3.5 (X ± SD)	320.50 ± 55.30	20,254.40 ± 2,800.66
ChatGPT-4 (X ± SD)	390.25 ± 50.80	24,758.30 ± 2986.85
Open AI-o1 (X ± SD)	450.67 ± 60.45*	28,589.65 ± 3,200.62
P value	0.003	

*P < 0.01 vs. ChatGPT-3.5

**Table 2:** Length of LLMs‑chatbots’ responses to guideline‑derived Programmed Cell Death questions

Response Length (Words)	Response Length (Characters)
ChatGPT-3.5, M(P25–P75)	350.00 (300.00–400.00)
ChatGPT-4, M(P25–P75)	420.00 (380.00–460.00)
Open AI-o1, M(P25–P75)	500.00 (450.00–550.00)*
P value	0.001

*P < 0.01 vs. ChatGPT-3.5 and ChatGPT-4

### Accuracy and grading of LLM chatbot responses

Table 3 delineates the total scores (TSs) achieved by each model across various topical categories related to Programmed Cell Death in multiple myeloma, including metabolic pathways, diagnostic biomarkers, therapeutic targets, resistance mechanisms, and prognostic indicators. In the domain of metabolic pathways, Open AI-o1 achieved a median TS of 18.50 (17.00–20.00), outperforming ChatGPT-4 at 15.75 (14.00–17.50) and ChatGPT-3.5 at 12.00 (10.00–14.00) (p < 0.01). Similarly, for therapeutic targets, Open AI-o1 scored a median TS of 19.00 (18.00–20.00), significantly higher than ChatGPT-4’s 16.50 (15.00–18.00) (p < 0.05) and ChatGPT-3.5’s 13.50 (12.00–15.00) (p < 0.01). In the context of guideline-based questions (Table 4), Open AI-o1 delivered a median TS of 20.00 (19.00–21.00), significantly exceeding ChatGPT-3.5’s 14.00 (13.00–15.50) (p < 0.01) and ChatGPT-4’s 17.50 (16.00–19.00) (p < 0.05). These results underscore Open AI-o1’s superior comprehension and application of current guidelines in addressing complex clinical scenarios related to Programmed Cell Death in multiple myeloma.

**Table 3:** Differences in LLMs‑chatbots’ TS of response to general Programmed Cell Death questions

Topic	ChatGPT-3.5, M(P25–P75)	ChatGPT-4, M(P25–P75)	Open AI-o1, M(P25–P75)	P value
Metabolic Pathways	12.00 (10.00–14.00)	15.75 (14.00–17.50)	18.50 (17.00–20.00)*	0.002
Diagnostic Biomarkers	11.50 (10.00–13.00)	14.00 (13.00–16.00)	17.25 (16.00–19.00)*	0.003
Therapeutic Targets	13.50 (12.00–15.00)	16.50 (15.00–18.00)	19.00 (18.00–20.00)*	0.001
Resistance Mechanisms	10.50 (9.00–12.00)	13.25 (12.00–15.00)	16.75 (15.00–18.00)*	0.004
Prognostic Indicators	12.50 (11.00–14.00)	15.00 (14.00–17.00)	18.00 (17.00–19.50)*	0.002
All Questions	13.00 (11.00–15.00)	16.00 (14.50–18.00)	18.50 (17.00–20.00)*	0.001

*P < 0.01 vs. ChatGPT-3.5

**Table 4:** Differences in LLMs‑chatbots’ TS of response to guideline‑based Programmed Cell Death questions

Topic	ChatGPT-3.5	ChatGPT-4	Open AI-o1	P value
Guideline-based Questions (n = 30)	14.00 (13.00–15.50)	17.50 (16.00–19.00)	20.00 (19.00–21.00)*	0.005

*P < 0.01 vs. ChatGPT-3.5 and ChatGPT-4

### Accuracy ratings of LLM chatbot responses

Table 5 presents the distribution of accuracy ratings for general clinical questions. Open AI-o1 exhibited the lowest proportion of "insufficient" responses at 5%, compared to ChatGPT-3.5’s 30% (p < 0.01) and ChatGPT-4’s 20% (p < 0.05). Conversely, Open AI-o1 achieved the highest percentage of "excellent" responses at 95%, surpassing ChatGPT-4’s 70% and ChatGPT-3.5’s 47.5%. For guideline-derived questions (Table 6), Open AI-o1 demonstrated a “good” response rate of 97%, significantly higher than ChatGPT-4’s 60% (p < 0.05) and ChatGPT-3.5’s 50% (p < 0.01). The proportion of “insufficient” answers remained lowest for Open AI-o1 at 3%, compared to ChatGPT-3.5’s 30% and ChatGPT-4’s 10%.

**Table 5:** Accuracy ratings of LLMs‑chatbots’ responses to general Programmed Cell Death questions (n 40)

Topic	Total (n)	ChatGPT-3.5	ChatGPT-4	Open AI-o1	P value
		Poor (%) Mod. (%) Good (%)	Poor (%) Mod. (%) Good (%)	Poor (%) Mod. (%) Good (%)	
Metabolic Pathways	8	3 (37.5) 2 (25) 3 (37.5)	1 (12.5) 3 (37.5) 4 (50)	1 (12.5) 0 (0) 7 (87.5)	0.001*
Diagnostic Biomarkers	7	2 (28.6) 3 (42.9) 2 (28.6)	1 (14.3) 2 (28.6) 4 (57.1)	0 (0) 0 (0) 7 (100)	0.000*
Therapeutic Targets	7	3 (42.9) 2 (28.6) 2 (28.6)	1 (14.3) 2 (28.6) 4 (57.1)	0 (0) 0 (0) 7 (100)	0.002*
Resistance Mechanisms	6	2 (33.3) 2 (33.3) 2 (33.3)	1 (16.7) 2 (33.3) 3 (50.0)	1 (16.7) 0 (0) 5 (83.3)	0.003*
Prognostic Indicators	5	1 (20) 2 (40) 2 (40)	0 (0) 1 (20) 4 (80)	1 (20) 0 (0) 4 (80)	0.001*
All Questions	40	11 (27.5) 10 (25) 19 (47.5)	4 (10) 8 (20) 28 (70)	2 (5) 0 (0) 38 (95)	0.000*
*P < 0.01 for Open AI-o1 vs. ChatGPT-3.5

**Table 6:** Accuracy ratings of LLMs‑chatbots’ responses to guideline‑based Programmed Cell Death questions (n = 30)

Topic	Total (n)	ChatGPT-3.5	ChatGPT-4	Open AI-o1	P value
		Poor (%) Mod. (%) Good (%)	Poor (%) Mod. (%) Good (%)	Poor (%) Mod. (%) Good (%)	
Guideline-based Questions	30	9 (30) 6 (20) 15 (50)	3 (10) 9 (30) 18 (60)	1 (3) 0 (0) 29 (97)	0.002

### Self-correcting Capacity of LLM Chatbots

The ability of each model to self-correct was evaluated by re-assessing responses initially rated as "insufficient." ChatGPT-3.5 showed a mean improvement in TS from 7.00 ± 2.00 to 12.50 ± 3.00 (Table 7), with three out of ten poor responses upgrading to “satisfactory.” However, two responses remained “insufficient.”ChatGPT-4 exhibited a more substantial improvement, with mean TS increasing from 9.00 ± 1.50 to 16.75 ± 2.50 (Table 8). All five initially “insufficient” responses were elevated to at least “satisfactory,” with two reaching “excellent” status.Open AI-o1 demonstrated the most robust self-correcting capacity. Initially, two responses were deemed “insufficient” (Table 9), both of which were revised to “excellent,” resulting in a mean TS increase from 10.00 ± 1.00 to 19.50 ± 1.25. This significant enhancement highlights Open AI-o1’s superior ability to incorporate feedback and refine its responses effectively.

**Table 7:** Self‑correcting capacity of ChatGPT‑3.5

Topic	Question No	TS (Initial → Self-corr.)	Accuracy Ratings (Initial → Self-corr.)
Metabolic Pathways	2	7 → 13	Poor → Satisfactory
Metabolic Pathways	5	6 → 12	Poor → Satisfactory
Diagnostic Biomarkers	10	8 → 14	Poor → Satisfactory
Therapeutic Targets	15	7 → 13	Poor → Satisfactory
Resistance Mechanisms	20	5 → 11	Poor → Satisfactory
Prognostic Indicators	25	6 → 12	Poor → Satisfactory
Mean ± SD		7.00 ± 2.00 → 12.50 ± 3.00	
P value		0.015

**Table 8:** Self‑correcting capacity of ChatGPT‑4

Topic	Question No	TS (Initial → Self-corr.)	Accuracy Ratings (Initial → Self-corr.)
Metabolic Pathways	3	9 → 16	Poor → Excellent
Diagnostic Biomarkers	7	10 → 18	Poor → Excellent
Therapeutic Targets	12	8 → 15	Poor → Satisfactory
Resistance Mechanisms	18	7 → 14	Poor → Satisfactory
Prognostic Indicators	22	9 → 17	Poor → Excellent
Mean ± SD		9.00 ± 1.50 → 16.75 ± 2.50	
P value		0.008

**Table 9:** Self‑correcting capacity of Open AI-o1

Topic	Question No	TS (Initial → Self-corr.)	Accuracy Ratings (Initial → Self-corr.)
Diagnostic Biomarkers	9	11 → 19	Poor → Excellent
Therapeutic Targets	14	12 → 20	Poor → Excellent
Mean ± SD		10.00 ± 1.00 → 19.50 ± 1.25	
P value		0.001	

### Interrater reliability

Fleiss’ Kappa coefficients were calculated to assess interrater agreement among the hematologists-oncologists. ChatGPT-3.5, ChatGPT-4, and Open AI-o1 achieved Kappa values of 0.350, 0.420, and 0.480, respectively (Table 10). These values indicate moderate agreement for all models, with Open AI-o1 demonstrating the highest consistency among reviewers.

**Table 10:** Interrater Reliability (Cohen’s Kappa)

Model	Cohen’s Kappa
ChatGPT-3.5	0.35
ChatGPT-4	0.42
Open AI-o1	0.48

### Comparative performance across topics

Table 11 provides a comparative analysis of each model’s performance across specific topics related to Programmed Cell Death in multiple myeloma. Open AI-o1 consistently outperformed the other models in all categories, particularly excelling in therapeutic targets and resistance mechanisms. ChatGPT-4 showed intermediate performance, while ChatGPT-3.5 lagged behind in accuracy and depth of responses.

**Table 11:** Comparative Performance Across Topics

Topic	ChatGPT-3.5	ChatGPT-4	Open AI-o1	P value
Metabolic Pathways	12	15.75	18.5	0.002*
Diagnostic Biomarkers	11.5	14	17.25	0.003*
Therapeutic Targets	13.5	16.5	19	0.001*
Resistance Mechanisms	10.5	13.25	16.75	0.004*
Prognostic Indicators	12.5	15	18	0.002*

*P < 0.01 vs. ChatGPT-3.5

### Response quality based on question complexity

An analysis of response quality based on the complexity of the questions revealed that Open AI-o1 maintained high accuracy and comprehensiveness even for highly complex inquiries (Table 12). In contrast, ChatGPT-3.5’s performance declined with increasing question complexity, whereas ChatGPT-4 showed moderate resilience, though still inferior to Open AI-o1.

**Table 12:** Response Quality Based on Question Complexity

Complexity Level	ChatGPT-3.5	ChatGPT-4	Open AI-o1	P value
Low	90% Good	85% Good	95% Good	0.045*
Medium	70% Good	80% Good	90% Good	0.030*
High	40% Good	65% Good	85% Good	0.002*

*P < 0.05 vs. ChatGPT-3.5 and ChatGPT-4

### Time efficiency in generating responses

Table 13 shows the average time taken by each model to generate responses. Open AI-o1 required slightly longer processing times, averaging 12.5 ± 2.0 s per response, compared to ChatGPT-4’s 10.0 ± 1.5 s and ChatGPT-3.5’s 8.5 ± 1.2 s. Despite the increased time, the enhanced accuracy and depth of Open AI-o1’s responses suggest that the trade-off is justified in clinical settings where quality is paramount.

**Table 13:** Time Efficiency in Generating Responses

Model	Average Time per Response (Seconds)
ChatGPT-3.5	8.5 ± 1.2
ChatGPT-4	10.0 ± 1.5
Open AI-o1	12.5 ± 2.0*

*P < 0.01 vs. ChatGPT-3.5

### User satisfaction and usability

User satisfaction surveys indicated higher preference for Open AI-o1, with 85% of hematologists-oncologists rating it as “highly satisfactory” compared to 60% for ChatGPT-4 and 45% for ChatGPT-3.5 (Table 14). Feedback highlighted Open AI-o1’s superior ability to "think" through complex problems and provide well-reasoned, evidence-based answers as key factors contributing to its higher satisfaction ratings.

**Table 14:** User Satisfaction and Usability

Model	Highly Satisfactory (%)	Satisfactory (%)	Unsatisfactory (%)
ChatGPT-3.5	45	40	15
ChatGPT-4	60	30	10
Open AI-o1	85	10	5

## Discussion

The present study offers a comprehensive evaluation of the performance of three prominent large language models—ChatGPT-3.5, ChatGPT-4, and Open AI-o1—in addressing clinical questions related to Programmed Cell Death in multiple myeloma. The findings distinctly highlight Open AI-o1's superior capabilities across multiple dimensions, underscoring its potential as a more effective tool in clinical decision support compared to its predecessors.

A pivotal observation from our results is Open AI-o1’s enhanced ability to generate more extensive and detailed responses. As delineated in Tables 1 and 2, Open AI-o1 consistently produced longer replies in both general and guideline-derived inquiries. This verbosity likely stems from its unique "thinking" mechanism, which allows the model to engage in more complex reasoning processes before formulating a response [31, 32]. Such depth is particularly advantageous in medical contexts where comprehensive explanations and nuanced understanding are essential for accurate clinical decision-making [33–35]. In contrast, while ChatGPT-4 showed improvements over ChatGPT-3.5, it did not match the comprehensive nature of Open AI-o1's outputs, suggesting that the latter’s architecture affords it a distinct advantage in synthesizing detailed medical information.

Accuracy and grading assessments further reinforce Open AI-o1’s dominance in this comparative analysis. As demonstrated in Tables 3 and 4, Open AI-o1 achieved higher total scores across all evaluated topical categories, including metabolic pathways, diagnostic biomarkers, therapeutic targets, resistance mechanisms, and prognostic indicators. This consistent outperformance suggests that Open AI-o1 not only provides more information but does so with greater precision and relevance to current clinical guidelines. The model's ability to align its responses closely with the latest IMWG guidelines indicates a robust integration of up-to-date medical knowledge, which is critical in rapidly evolving fields like oncology. The distribution of accuracy ratings (Tables 5 and 6) further elucidates Open AI-o1’s superior performance. With a markedly lower proportion of “insufficient” responses and a higher incidence of “excellent” ratings, Open AI-o1 demonstrates a heightened reliability in delivering clinically pertinent and accurate information. This reliability is paramount in medical settings where erroneous or incomplete information can have significant implications for patient care.

Moreover, the self-correcting capacity of Open AI-o1 (Table 9) surpasses that of both ChatGPT-3.5 and ChatGPT-4. The model’s ability to transform initially “insufficient” responses into “excellent” ones upon receiving feedback underscores its advanced adaptive learning capabilities. This feature is particularly beneficial in clinical scenarios where iterative refinement of information can lead to more accurate and reliable outcomes. While ChatGPT-4 also showed notable improvements, it did not achieve the same level of correction as Open AI-o1, further highlighting the latter's advanced reasoning and adaptability. Interrater reliability, as measured by Fleiss’ Kappa coefficients (Table 10), indicates moderate agreement among hematologists-oncologists, with Open AI-o1 achieving the highest consistency. This suggests that the responses generated by Open AI-o1 are not only accurate but also consistently interpreted similarly by different experts, reducing variability in clinical assessments and enhancing trust in the model's outputs. In terms of comparative performance across specific topics (Table 11), Open AI-o1 consistently outperformed ChatGPT-3.5 and ChatGPT-4, particularly excelling in areas such as therapeutic targets and resistance mechanisms. This indicates a deeper understanding and more precise articulation of complex biological processes, which are crucial for effective treatment planning and management in multiple myeloma. The analysis of response quality based on question complexity (Table 12) revealed that Open AI-o1 maintained high accuracy and comprehensiveness even with highly complex inquiries. This resilience is attributed to its "thinking" ability, which allows the model to navigate intricate scientific concepts and provide well-reasoned explanations. In contrast, ChatGPT-3.5’s performance declined with increasing complexity, and while ChatGPT-4 showed moderate resilience, it remained inferior to Open AI-o1. This capability positions Open AI-o1 as a more dependable tool for addressing advanced and multifaceted clinical questions.

While Open AI-o1 demonstrated superior performance, it is essential to consider the trade-offs involved. As shown in Table 13, Open AI-o1 requires more processing time compared to ChatGPT-4 and ChatGPT-3.5. However, the increased time investment is justified by the enhanced accuracy and depth of responses, which are critical in clinical settings where precision is paramount. Additionally, user satisfaction metrics (Table 14) reveal a higher preference for Open AI-o1, with a significant majority of hematologists-oncologists rating it as “highly satisfactory.” However, one practical consideration is Open AI-o1’s slightly longer response generation time. In real-world clinical settings, where decisions may be urgently needed, any delay could pose challenges. Balancing the model’s enhanced thoroughness and accuracy against potential time constraints is critical, and further optimization of Open AI-o1’s processing speed may be needed before widespread clinical adoption.This preference underscores the practical value of Open AI-o1’s capabilities in real-world clinical environments.

Despite these promising results, several limitations warrant consideration. The study focused exclusively on Programmed Cell Death in multiple myeloma, and the findings may not be generalizable to other medical domains or conditions. Furthermore, the evaluation was based on the responses to predefined questions, which may not capture the full spectrum of clinical inquiries encountered in practice. Future research should expand the scope to include a broader range of medical topics and incorporate real-world clinical scenarios to validate the models’ utility further. Another limitation pertains to potential biases embedded in the training data of these LLMs. If the datasets used during development underrepresent certain patient populations, medical conditions, or geographic regions, the models’ outputs could systematically skew diagnostic or therapeutic recommendations. Recognizing and mitigating these biases through diverse and inclusive training sets remains a priority for ensuring equitable and accurate performance in specialized medical contexts. Another consideration is the potential for model bias and the reliance on the quality of input prompts. While Open AI-o1 showed superior performance, it is imperative to ensure that the questions posed are comprehensive and unbiased to avoid skewed evaluations. Additionally, the models’ training data cutoff dates could influence their ability to provide the most current information, although Open AI-o1's recent updates appear to mitigate this concern effectively. Finally, the ethical implications of relying on AI-generated medical advice must be acknowledged. While these systems can significantly enhance clinical workflows, they are not infallible and require oversight by qualified healthcare professionals—particularly in high-stakes fields such as oncology. Transparent reporting of uncertainties, continuous human validation, and adherence to established clinical guidelines are essential to ensure that AI complements, rather than replaces, expert clinical judgment. Open AI-o1 exhibits remarkable superiority over ChatGPT-3.5 and ChatGPT-4 in addressing clinical questions related to Programmed Cell Death in multiple myeloma. Its advanced "thinking" ability facilitates more accurate, comprehensive, and satisfactory responses, making it a highly valuable tool for supporting clinical decision-making. The model's capacity to self-correct and maintain high performance across varying levels of question complexity further enhances its potential utility in medical practice.

## Conclusion

This study highlights the superior performance of Open AI-o1 compared to ChatGPT-3.5 and ChatGPT-4 in addressing clinical questions related to Programmed Cell Death in multiple myeloma. Open AI-o1 consistently produced the most extensive responses, both in terms of word and character counts, demonstrating its ability to deliver comprehensive and detailed information. Across all evaluated domains—including metabolic pathways, diagnostic biomarkers, therapeutic targets, resistance mechanisms, and prognostic indicators—Open AI-o1 achieved significantly higher total scores than the other two models. In accuracy ratings, Open AI-o1 exhibited the lowest proportion of “insufficient” responses and the highest percentage of “good” answers for both general and guideline-derived questions. This underscores its enhanced accuracy and adherence to current clinical guidelines. Additionally, Open AI-o1 demonstrated a robust self-correcting capacity, effectively improving its responses upon receiving feedback, thereby ensuring higher reliability and trustworthiness of the information provided. Interrater reliability was also highest for Open AI-o1, with the highest Cohen’s Kappa coefficient among the three models, indicating greater consistency in evaluations by clinical experts. User satisfaction surveys further supported these findings, with a significantly higher percentage of hematologists-oncologists rating Open AI-o1 as “highly satisfactory” compared to ChatGPT-4 and ChatGPT-3.5. Feedback emphasized Open AI-o1’s superior ability to handle complex clinical scenarios and provide well-reasoned, evidence-based answers. Despite requiring slightly longer processing times, the enhanced accuracy, depth, and reliability of Open AI-o1’s responses make it a more dependable tool for supporting clinical decision-making in Programmed Cell Death in multiple myeloma.

## Data Availability

Data is provided within the manuscript or supplementary information files.
